# Antidiabetic Effect of Substituting Processed Meat with Reduced-Fat and Diatomaceous Earth-Enriched Pâtés in Middle-Aged Female Wistar Rats

**DOI:** 10.3390/foods15050878

**Published:** 2026-03-04

**Authors:** Rocío Redondo-Castillejo, Claudia Quevedo-Torremocha, María Luisa de la Cruz Conty, Marina Hernández-Martín, Aránzazu Bocanegra, Adrián Macho-González, Susana Cofrades, María Dolores Álvarez, Sara Bastida, María Elvira López-Oliva, Juana Benedí, Alba Garcimartín

**Affiliations:** 1 Pharmacology, Pharmacognosy and Botany Department, Pharmacy School, Complutense University of Madrid, 28040 Madrid, Spain; roredond@ucm.es (R.R.-C.); clauquev@ucm.es (C.Q.-T.); jbenedi@ucm.es (J.B.); a.garcimartin@ucm.es (A.G.); 2 AFUSAN Research Group, Sanitary Research Institute of the San Carlos Clinical Hospital (IdISSC), 28040 Madrid, Spain; marinh04@ucm.es (M.H.-M.); sbastida@ucm.es (S.B.); elopez@ucm.es (M.E.L.-O.); 3 Department of Statistics and Operations, Medicine School, Complutense University of Madrid, 28040 Madrid, Spain; ml.cruz@ucm.es; 4 Departmental Section of Physiology, Pharmacy School, Complutense University of Madrid, 28040 Madrid, Spain; 5 Nutrition Unit, Nutrition and Food Science Department, Pharmacy School, Complutense University of Madrid, 28040 Madrid, Spain; 6 Institute of Food Science, Technology and Nutrition (ICTAN), Spanish National Research Council (CSIC), 28040 Madrid, Spain; scofrades@ictan.csic.es (S.C.); mayoyes@ictan.csic.es (M.D.Á.)

**Keywords:** functional food, silicon, biopolymeric emulsion, female rats, nutritional intervention

## Abstract

This study evaluates a non-invasive and feasible nutritional strategy as a realistic intervention to prevent or mitigate T2DM in one-year-old female Wistar rats. This strategy is based on replacing a commercial pâté (CP) with a functional one, either a silicon-enriched commercial pâté (Si-CP), a reduced-fat pâté formulated with a biopolymeric emulsion (BP), or a silicon-enriched and reduced-fat biopolymeric pâté (Si-BP). After consumption of a high-saturated fat high-cholesterol diet, CP rats exhibited elevated fecal excretion, fasting serum glucose, insulin, and LDL cholesterol, and altered islet morphology. Versus the CP group, the Si-CP consumption group exhibited significantly reduced fecal output (1.17 ± 0.02 vs. 2.09 ± 0.44) and serum insulin (12.06 ± 7.89 vs. 20.74 ± 7.44), triglycerides (47.51 ± 4.46 vs. 58.24 ± 9.97), LDL cholesterol (34.63 ± 5.14 vs. 42.20 ± 4.98), and ghrelin (32.49 ± 24.66 vs. 78.35 ± 22.85). Although BP rats also exhibited some positive effects, Si-BP animals presented the most promising results. Compared to the CP group, Si-BP consumption significantly reduced fecal excretion (1.44 ± 0.24) and serum glucose (129.1 ± 10.40 vs. 154.9 ± 15.76), insulin (9.49 ± 6.06), triglycerides (46.91 ± 5.13), and estradiol (528.2 ± 45.00 vs. 634.4 ± 98.87), preserved islet circularity (0.88 ± 0.02 vs. 0.82 ± 0.01), and significantly increased tibia length (4.09 ± 0.12 vs. 3.95 ± 0.09) and wet weight (0.65 ± 0.07 vs. 0.56 ± 0.06). This study demonstrates the antidiabetic effects of silicon from diatomaceous earth (4 mg Si/kg body/day) incorporated into pâté in middle-aged female rats. Replacing CP with a functional alternative improved the health status of diabetic female rats, supporting its potential as an effective nutritional adjuvant.

## 1. Introduction

Type 2 diabetes mellitus (T2DM) is a chronic metabolic disorder with a prevalence of more than 6% of the global population, representing one of the main health challenges worldwide. Lifestyle factors such as sedentarism and unbalanced diets contribute to T2DM development, which worsens with ageing. Additionally, sex-specific biological and hormonal differences should be considered when evaluating T2DM risk and outcomes, since the existing evidence is limited [1]. Although T2DM is primarily characterized by insulin resistance, it occurs within the framework of metabolic syndrome, alongside profound metabolic dysregulation, oxidative stress, and low-grade inflammation. These metabolic disturbances usually result in elevated triglycerides and cholesterol levels, increasing cardiovascular risk. Simultaneously, T2DM promotes hormonal alterations, affecting appetite modulation, energy expenditure, and systemic metabolic homeostasis. Hence, parameters such as ghrelin and insulin-like growth factor 1 (IGF-1) are generally altered in T2DM rodent models, modifying satiety control, insulin secretion and sensitivity, and lipid metabolism [2,3]. Due to the protective role of estrogens against weight gain, insulin resistance, cardiovascular risk, and bone fragility, their analysis in female rats is particularly useful. Therefore, the simultaneous assessment of glucose and insulin levels, lipid parameters, and key hormones is necessary for an integrative approach to T2DM.

The consumption of high-fat Western diets is considered a major risk factor in the onset of T2DM. A notable component of these diets is high meat intake, particularly processed meat, which can significantly contribute to saturated fat intake, caloric density, and deleterious metabolic effects [4,5]. However, meat products are also sources of valuable nutrients and, due to their widespread consumption, specifically among T2DM patients, they can be used as suitable matrices for developing functional foods [6,7]. Among technological strategies, reducing both the amount and bioavailability of fat has gained increasing interest [8,9]. It has been demonstrated that emulsions formulated with lard and stabilized by various biopolymers are an effective technological approach to reduce both fat content and lipid digestibility. Another strategy to develop functional meat products involves the incorporation of bioactive compounds into the meat matrix [10]. In this regard, silicon emerges as a promising candidate because of its antidiabetic and lipid-lowering properties. Evidence indicates that silicon, consumed as part of a functional meat product, exerts hypoglycemic, hypocholesterolemic and hypotriglyceridemic effects in male rats with metabolic syndrome and late-stage T2DM [11,12]. Although the effects of silicon on diabetic females have not yet been investigated, recent findings demonstrate an interesting lipid-lowering effect in healthy female rats [13]. Moreover, it can be highlighted that both strategies for developing functional foods (biopolymer-based emulsions and silicon enrichment) may be applied simultaneously. Previous studies have demonstrated that silicon-enriched biopolymeric emulsions present suitable technological properties and stability during chilled storage. Furthermore, these silicon-enriched biopolymeric emulsions reduce fat digestion, making them potential candidates for the development of healthier meat products [14,15]. Nevertheless, the in vivo hypolipemic efficacy of silicon-enriched biopolymeric emulsions when they are included in a meat matrix and consumed as a functional food remains unexplored.

Based on results, the present study hypothesizes that the consumption of pâtés with reduced fat content, impaired lipid digestibility, and silicon enrichment slow down T2DM progression. Hence, the objective of this work is to evaluate the consequences of substituting a commercial pâté with a functional one in the frame of a high-saturated-fat high-cholesterol diet (HSFHCD) in female rats with T2DM. To reach this objective, we evaluated the effects of consuming three different functional pâtés [silicon-enriched (Si-CP), fat-reduced (BP), and fat-reduced and silicon-enriched (Si-BP)] on (a) dietary intake, fecal excretion, and the digestibility of both the diet and silicon; (b) weight gain and relative weight of selected organ relevant to metabolic regulation; (c) serum glucose and insulin concentrations; (d) serum cholesterol, triglycerides, and lipoproteins; (e) serum ghrelin, IGF-1, and estradiol levels; and (f) bone parameters, in female rats with T2DM induced by a HSFHCD. This simple but novel integrative approach may help demonstrate the utility of functional meat products, identify potential mechanisms for T2DM management, and delay the onset of long-term complications associated with the disease.

## 2. Materials and Methods

### 2.1. Materials and Chemicals

Lard (99.9% fat content) was obtained through clarification of Iberian pork fat, which, together with lean pork meat and pork liver, was purchased from a local supermarket in Madrid, Spain. A pork rind protein extract (PRP) containing 90% protein (70% of which was collagen) and 10% fat was sourced from Prosur (Murcia, Spain). Microbial transglutaminase (MTG) (Activa^®^ EB) with an activity of 50 U/g was kindly donated by Ajinomoto Foods Europe (Paris, France). κ-carrageenan (κC) was supplied by Tradissimo, Trades S.A. (Barcelona, Spain). A food-grade preparation of diatomaceous earth powder (DE; Tierra de Diatomeas^®^), with a silicon dioxide (SiO_2_) content of 85% (equivalent to 40% elemental Si), was generously provided by Vitality Gesf S.L. (Valencia, Spain).

### 2.2. Preparation of Oil-Water Emulsions

Oil-in-water emulsions (O/W 40/60) were prepared using a two-step emulsification process following the protocol described by Cofrades et al. [15]. First, the PRP (10.86% *w*/*v*) was hydrated with half of the water from the aqueous phase. The structuring solution, containing 3.62% (*w*/*v*) MTG and 2.71% (*w*/*v*) κC, was prepared separately using the remaining water. For the silicon-enriched biopolymeric emulsion, 1.71% (*w*/*w*) DE was added to the melted lard and stirred for 30 min. Then, a primary emulsion was produced by homogenizing the PRP solution with melted lard, with or without DE, at 13,200 rpm for 2 min using a homogenizer (Ultraturrax, IKA-25, Staufen, Germany). The pH was adjusted to 7.0 using 1 M hydrochloric acid (HCl). This primary emulsion was stored for 1 h to allow the protein coating of fat droplets. After that, the structuring solution was added to the primary emulsion and mixed using a rod stirrer (Bunsen AGV-8, Madrid, Spain) at 500 rpm for 1 min until fully homogenized, yielding the PRP (3%)/MTG (1%)/κC (0.75%) emulsion. Aliquots of 10 g were quickly poured into cylindrical containers (3.5 cm height × 2.5 cm diameter) with lids and stored at 4 ± 2 °C for 72 h to allow the emulsions to set prior to pâté preparation.

### 2.3. Preparation of Pâtés

Four pâtés were specifically formulated, differing in their fat and silicon content. Detailed composition is shown in Table 1. The so-called commercial pâté (CP) was formulated to resemble the traditional marketed products, with a typical fat content of ~30%. The silicon-enriched commercial pâté (Si-CP) was prepared by adding 0.44% (*w*/*w*) DE to the lard prior to mixing, ensuring a final silicon concentration of 0.05% (*w*/*w*) in the product. The biopolymeric pâté (BP) was formulated with lard partially replaced with the biopolymeric (PRP/MTG/κC/) emulsion, resulting in a reduced-fat pâté (~15% of fat) with impaired lipid digestibility. The silicon-enriched BP (Si-BP) was prepared using the same procedure as for the BP, but employing the silicon-enriched biopolymeric emulsion, also resulting in a final silicon concentration of 0.05% (*w*/*w*). The preparation of the different pâtés followed the method described by Cofrades et al. [16]. Briefly, meat, liver, and lard were thawed before use (at 2 ± 2 °C for 24 h). The chilled meat was placed in a food processor (Thermomix, Vorwerk, Wuppertal, Germany) and processed at speed setting 4 for 4 min. The liver was then added and mixed for an additional 2 min. After that, the fat source (either lard or emulsion) was incorporated and homogenized for 4 min. Next, half of the additives (i.e., 1 g sodium chloride/100 g, 1 g milk powder/100 g, 0.5 g sodium caseinate/100 g, 0.25 g sodium tripolyphosphate/100 g and 0.16 g flavoring/100 g) were added (Table 1). The mixture was homogenized again for 1 min, followed by the addition of water and mixing for 1 more minute. Finally, the remaining half of the additives were incorporated, and the mixture was homogenized at speed 7 until a uniform batter was obtained. The total processing time was approximately 12 min. The batters were divided into aliquots and placed in sealed bottles, then heated in a water bath at 85 °C until the thermal center reached 80 °C (~25 min). After heat processing, the pâtés were cooled in a water bath (15–18 °C) and stored under refrigeration (3 ± 1 °C) for no longer than 2 weeks until use. They were discarded if organoleptic alterations, such as changes in color or odor, were detected.

### 2.4. Experimental Design

Twenty-eight four-month-old female Wistar rats were obtained from Harlan S.L. (Barcelona, Spain) and reared until reaching one year old. They were housed in the Animal Experimentation Center of Alcalá University, Madrid, Spain (registration number ES280050001165). The experimental protocol was approved by the Spanish Science and Technology Advisory Committee (PROEX 315.2/21). All procedures complied with Directive 2010/63/EU on the protection of animals used for scientific purposes. In addition, the study adhered to the ARRIVE guidelines to ensure ethical standards and transparency in animal research. Rats were housed in pairs or trios to minimize social stress, under standard conditions (22 ± 2 °C, 55 ± 10% relative humidity, 12-h light/dark cycle) with free access to maintenance diet and tap water and provided with environmental enrichment. Once rats were one year old, they were randomly assigned to four experimental groups (*n* = 7, group-housed in three cages; two cages with two rats each and one cage with three rats), ensuring similar initial fasting glucose levels. All groups were fed a HSFHCD ad libitum, U8958P version 370, purchased from Safe (Augy, France). The detailed composition of the diet is provided in Supplementary Table S1. Basal diet intake was recorded at the cage level by weighing feeders every 24 h (pre- and post-weighing) and by collecting and weighing any spillage from cage liners to correct the daily intake. Additionally, each morning, animals were individually transferred to their own clean cage, where they ate 3 g of their assigned experimental pâté. The Si-CP and Si-BP formulations supplied a daily dose of 4 mg Si/kg body weight. Full consumption of the pâté was confirmed within 1 h before the animals were returned to their home cage, thereby ensuring individual attribution of pâté intake. For descriptive purposes, per-animal basal diet intake was derived by calculating cage-level intake divided by the number of occupants. Body weight and food intake were recorded weekly throughout the 10-week experimental period. Serum glucose was measured at the baseline, at week 6, and in the final week of the study. In addition, vaginal smears were performed every other day during the final week to ensure that sacrifice occurred during the diestrus phase (Supplementary Figure S1). At the end of the experimental period, animals were fasted overnight before being sacrificed under isoflurane anesthesia by blood extraction from the descending aorta. Tissues and organs were isolated, washed with cold 0.9% saline solution, weighed, and immediately frozen at −80 °C or embedded in paraffin until further analysis. Relative organ weights were calculated by dividing absolute organ weight by body weight.

### 2.5. Fecal Excretion and Moisture Determination

The total amount of feces accumulated in the cage bedding was collected daily during the last week of the experiment. Individual fecal excretion was derived from cage feces divided by the number of occupants. Fecal samples were dried at 100 °C and fecal moisture (%) was calculated as follows:

(Wet weight (g) − Dry weight (g))/Wet weight (g) × 100.(1)

### 2.6. Determination of Silicon Content in Food, Water, and Feces

The silicon content in food, water, and fecal samples was analyzed. Food and feces were calcined in a muffle furnace at 500 °C for 5 h. Then, ashes were digested with a mixture of nitric acid (HNO_3_), hydrogen peroxide (H_2_O_2_), and hydrofluoric acid (HF) in a high-pressure microwave to ensure complete matrix dissolution. Water was filtered by 0.45 µm pore size for particle removal. Silicon was quantified by inductively coupled plasma optical emission spectrometry (ICP-OES) (CAI of Earth Sciences and Archaeometry, Faculty of Geological Sciences, UCM, Madrid, Spain). The analytical procedure was validated using bone ash as certified standard reference material (SRM 1400, NIST, Gaithersburg, MD, USA). All measurements were conducted in triplicate to ensure precision and reproducibility.

### 2.7. Digestibility Coefficients

Diet apparent digestibility (%) was calculated as follows:

(Feed intake (g) − Fecal output (g))/Feed intake (g) × 100,(2)

where total feed intake includes the sum of diet intake plus the 3 g of the experimental pâté.

Silicon digestibility (%) was calculated as follows:

(Silicon intake (mg) − Fecal silicon (mg))/Silicon intake (mg) × 100,(3)

where silicon intake includes the silicon provided by the diet, pâté and water, adjusted by consumption. Fecal silicon was determined by multiplying fecal output by its silicon concentration.

### 2.8. Pancreatic Islet Morphology

Pancreatic islet morphology was evaluated on hematoxylin and eosin-stained sections obtained from three different depths to ensure representative sampling. Images were captured at 100× using an Olympus BX50 optical microscope with a DP50 camera (Olympus, Madrid, Spain) and processed with ImageJ version 1.54g (NIH, Bethesda, MD, USA). Two researchers, blinded to the experimental groups, measured the total pancreas area (µm^2^), and the area (µm^2^) and perimeter (µm) of each islet. Islet areas were further classified into tertiles, sorted from smallest to largest, to obtain the percentage of islet within each tertile. The percentage of area covered by islet was determined as follows:

Sum islets areas/Pancreas area × 100.(4)

Finally, islet circularity was calculated as follows:

4π × Islet area/Islet perimeter^2^, (5)

where values close to 1 denote a shape approaching a perfect sphere [17].

### 2.9. Bone Moisture, Ash and Mineral Content Analysis

Tibias were isolated, measured, and weighed. Then, they were desiccated for 72 h at 100 °C. Bone moisture (%) was calculated as follows:

(Wet weight (g) − Dry weight (g))/Wet weight (g) × 100.(6)

Tibias were calcined at 650 °C for 10 h, and the resulting bone ashes were weighed. The mineral content was calculated as follows:

Ash weight (g)/Bone weight (g) × 100.(7)

Ashes were digested with a mixture of HNO_3_ and HCl in an oven at 100 °C for 24 h. The calcium, phosphorus, and magnesium contents were analyzed by ICP-OES (CAI of Earth Sciences and Archaeometry, Faculty of Geological Sciences, UCM, Madrid, Spain). The procedure was validated using an internationally certified reference material. All determinations were performed in triplicate to ensure precision and reproducibility.

### 2.10. Serum Determinations

Blood samples were centrifuged twice at 1500 *g* for 10 min to obtain serum. After deep-freeze storage for less than three months prior to analysis, the serum concentrations of glucose, cholesterol, triglycerides, insulin, estradiol, ghrelin, and IGF-1 were quantified, and lipoproteins were isolated. Serum glucose and cholesterol were determined using colorimetric kits (Spinreact, Barcelona, Spain). Insulin was measured using a rat insulin enzyme-linked immunosorbent assay (ELISA) kit (ELR-Insulin, RayBiotech, Inc., Peachtree Corners, GA, USA). The Homeostatic Model Assessment of Insulin Resistance (HOMA-IR) was calculated as follows:

Glucose (mmol/L) × Insulin (mIU/L)/22.5.(8)

Estradiol, ghrelin, and IGF-1 were quantified with the ELISA kits ER1507, ER1605 and ER0030 (FineTest, Wuhan, China). Lipoproteins were isolated via ultracentrifugation (Sorvall MX 120+ with S140-AT rotor, Thermo Scientific, Waltham, MA, USA) following a four-step protocol provided by Thermo Fisher Scientific Inc. [18]. Minor modifications in salt solutions and ultracentrifugation times were made to adapt it for rat samples, in accordance with Terpstra et al. [19]. Cholesterol was quantified in the lipoprotein fractions using a colorimetric kit (Spinreact, Barcelona, Spain). Atherogenic index was calculated as follows:

log_10_(Triglycerides/High-Density Lipoprotein cholesterol). (9)

### 2.11. Statistical Analysis

Descriptive analyses were first performed for all quantitative variables, with results reported as mean ± standard deviation. To obtain robust estimates of mean differences and 95% confidence intervals, a bootstrap procedure with 1000 resamples was applied using the boot package in R software (version 4.2.2) (Table S2). Subsequently, a one-way ANOVA was fitted to compare groups, followed by Tukey’s post hoc tests. Finally, the Pearson product–moment correlation coefficients were calculated to assess associations between fat and silicon intake and the other measured variables. *p* < 0.05 was considered statistically significant. Graphs were generated with GraphPad Prism version 8 (GraphPad software, Inc., La Jolla, CA, USA).

## 3. Results

### 3.1. Feed Consumption Characterization, Water Intake, Fecal Excretion, and Digestibility Parameters

**Figure 1:**
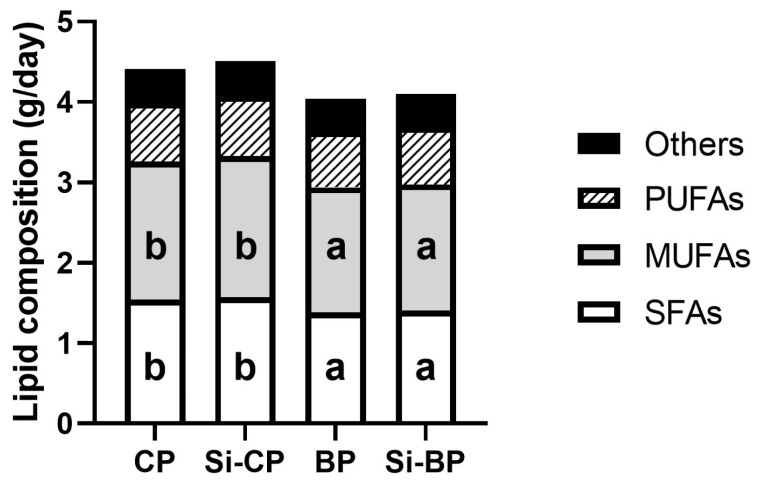
Daily lipid intake and fatty acid composition. Different letters (a < b) indicate statistically significant differences among groups (95% CIs of the mean differences not crossing zero, bootstrap procedure with 1000 resamples, one-way ANOVA, and Tukey’s post-hoc test). SFAs: saturated fatty acids; MUFAs: monounsaturated fatty acids; PUFAs: polyunsaturated fatty acids.

**Figure 2:**
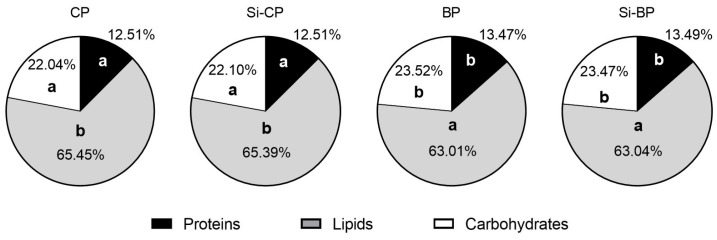
Macronutrient-derived energy of total daily intake. Different letters (a < b) indicate statistically significant differences among groups (95% CIs of the mean differences not crossing zero, bootstrap procedure with 1000 resamples, one-way ANOVA, and Tukey’s post-hoc test).

Table 2 summarizes the parameters related to feed and water consumption, fecal excretion, and digestibility recorded throughout the experiment. The total daily intake corresponded to the HSFHCD plus 3 g of the experimental pâté. Diet consumption did not differ significantly among groups, with mean daily intake ranging between 12.54 and 12.88 g/day. Total protein and carbohydrate intakes were also similar among groups (1.90 to 1.97 g/day and 3.35 to 3.44 g/day, respectively). In contrast, lipid consumption was significantly lower in the BP and Si-BP groups than in their CP and Si-CP counterparts, without changes in daily cholesterol intake. Regarding lipid composition of total daily intake (Figure 1), although intake of polyunsaturated fatty acids (PUFAs) was similar among groups, intakes of both saturated fatty acids (SFAs) and monounsaturated fatty acids (MUFAs) were also significantly lower in the BP and Si-BP groups than in the CP and Si-CP ones. Mean energy intake ranged from 57.75 to 62.07 kcal/day, without differences among groups. However, there were differences in the energy provided by macronutrients (Figure 2). Thus, energies derived from proteins and carbohydrates were significantly higher in the BP and Si-BP groups than in their CP and Si-CP peers. Subsequently, both the BP and Si-BP groups presented lower energy derived from lipids than CP and Si-CP ones. Finally, water intake was similar in all experimental groups (17.43 to 20.86 mL/day).

The consumption of experimental pâtés significantly affected fecal output (Supplementary Figure S2). Compared with the CP group, fecal excretion was lower in the Si-CP (−44.02%), BP (−34.45%) and Si-BP (−31.10%) groups. Si-BP rats exhibited fecal output similar to their BP peers and higher than that of the Si-CP animals. Fecal moisture remained unchanged across groups (16.58 to 18.90%). Si-CP, BP, and Si-BP rats exhibited significantly higher diet digestibility than CP ones (+9.89%, +8.44% and +8.38%, respectively). However, dietary apparent digestibility in Si-BP animals did not exceed that in the BP group, and both values were lower than in the Si-CP group.

Silicon intake was significantly higher in the Si-CP and Si-BP groups, by +49.70% compared with the BP and CP groups. Silicon excretion was lower in the Si-CP and BP groups relative to the CP group (−42.66% and −49.65%, respectively), while Si-BP rats showed equivalent silicon excretion to their CP counterparts. Furthermore, marked differences were observed in silicon digestibility, with all groups being significantly different from each other. CP animals showed negative values (−44.55%), whereas silicon digestibility increased up to 27.16% in the BP group, and even more in the Si-CP group (44.46%), although it increased up to 7.71% in the Si-BP group.

### 3.2. Ponderal Parameters and Organ Weights

**Figure 3:**
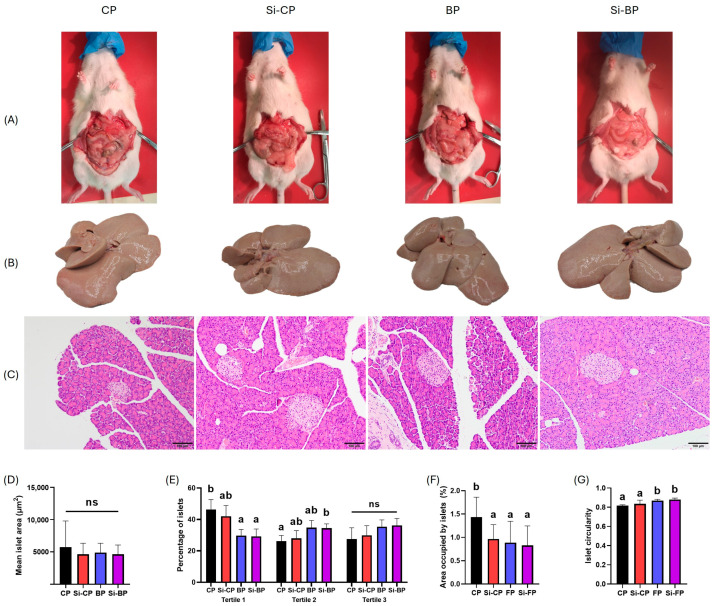
Representative macroscopic and histological features of rats fed different pâté formulations. Representative images of abdominal fat accumulation (**A**), macroscopic liver appearance (**B**), and pancreas histology (**C**) in rats from the CP, Si-CP, BP, and Si-BP groups. Panels (**C**–**G**) display histological sections of pancreatic tissue (hematoxylin and eosin staining) and quantitative analyses: (**D**) mean islet area, (**E**) islet size distribution (tertiles 1–3), (**F**) total pancreatic area occupied by islets, and (**G**) islet circularity. Scale bars = 100 μm. Different letters (a < b) indicate statistically significant differences among groups (95% CIs of the mean differences not crossing zero, bootstrap procedure with 1000 resamples, one-way ANOVA, and Tukey’s post-hoc test).

Table 3 shows the final body weight of rats, their body weight gain, and the relative weights of different white fat depots, livers, and pancreases. Figure 3 presents representative images of abdominal fat (A), livers (B), pancreases (C), and their histological parameters (D-G). There were no significant differences in the final body weight among groups, ranging from 361.4 to 391.6 g. However, body weight gain throughout the experiment varied depending on dietary treatment. Thus, there were no differences between CP and any of the other groups, although rats in the BP and Si-BP groups gained significantly less weight than those in the Si-CP one. The relative weights of gonadal, retroperitoneal, and subcutaneous fats depots, as well as livers, were similar across all experimental groups. Regarding pancreatic parameters, the relative pancreas weight showed a slight trend toward higher values in BP and Si-BP animals, compared with CP rats, and was significantly higher in the Si-CP ones. No significant differences were found among the silicon-enriched or fat-reduced groups. Moreover, with respect to pancreatic islet morphology, no differences were detected in the mean area of islets (Figure 3D), although their size distribution varied across experimental groups (Figure 3E). Thus, CP animals presented the highest percentage of islets (46.39%) whose area was in tertile 1 (i.e., the smallest). This figure tended to be lower in Si-CP rats and reached significance in BP and Si-BP groups (29.79% and 29.29%, respectively). CP rats had the lowest percentage of islets (26.12%) within tertile 2 (i.e., medium size), which tended to be higher in Si-CP and BP animals, and reached statistical significance in Si-BP ones (34.47%). In contrast, no differences were detected in the percentage of islets within tertile 3 (i.e., the largest), ranging from 27.49% in the CP group to 34.77% in BP one. Furthermore, the pancreas area covered by islets also differed among the experimental groups (Figure 3F). Hence, CP animals displayed significantly higher values than Si-CP, BP, or Si-BP rats, which were similar among themselves. Finally, the islets’ circularity was significantly higher in BP and Si-BP rats than in their CP and Si-CP peers (Figure 3G).

### 3.3. Serum Biochemical and Hormonal Parameters

Table 4 summarizes the effects of the different dietary interventions on metabolic and hormonal serum parameters. Initial and intermediate fasting serum glucose levels did not differ among groups; all means were lower than 100 mg/dL, consistent with non-established T2DM. At the end of the experiment, fasting glucose values exceeded 126 mg/dL in all experimental groups, although hyperglycemia severity differed depending on the experimental pâté consumed. Si-BP animals exhibited lower serum glucose than the other groups; meanwhile, insulin tended to be lower in BP and was significantly lower in the Si-CP (−41.85%) and Si-BP (−54.24%) groups than in the CP one. Thus, BP animals tended to have a lower HOMA-IR, whereas Si-CP (−42.21%) and Si-BP (−61.13%) rats showed a significant reduction in HOMA-IR relative to their CP counterparts. BP animals also showed a tendency toward lower levels of triglycerides, which reached significance in Si-CP (−18.42%) and Si-BP (−19.45%) animals compared with CP ones. Moreover, although total cholesterol did not differ among groups, its distribution within lipoproteins was altered. Si-BP rats tended to have lower very low-density lipoprotein (VLDL) cholesterol than their CP and BP peers, and significantly lower levels than those displayed by Si-CP rats. No differences were observed in intermediate-density lipoprotein (IDL) cholesterol among groups. However, low-density lipoprotein (LDL) cholesterol was lower in Si-CP animals and higher in Si-BP ones than in their CP and BP counterparts. Finally, Si-BP rats also presented higher HDL cholesterol than the rest of the animals, resulting in a significantly lower atherogenic index than the other groups. Regarding hormonal markers, serum ghrelin tended to be lower in BP and Si-BP rats and was significantly lower in Si-CP (−58.53%) rats compared with CP ones. Moreover, no significant changes were observed in serum IGF-1 levels in any experimental group with respect to CP, although these levels tended to increase in both Si-CP and BP groups, while tending to decrease in Si-BP one. In fact, Si-BP rats had significantly lower IGF-1 levels than the BP and Si-CP groups. Lastly, estradiol concentrations did not differ between the BP and CP groups, tended to decrease in the Si-CP group, and were significantly lower in the Si-BP group (−16.74%) in comparison with the CP one and compared with BP animals.

### 3.4. Bone Morphometric and Compositional Parameters

**Figure 4:**
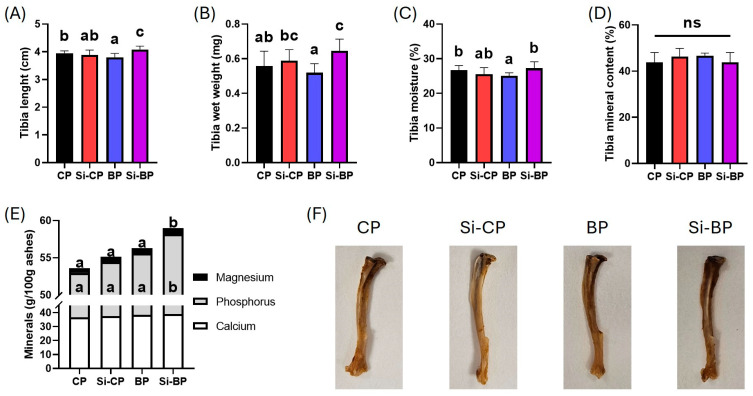
Tibia morphometric and compositional parameters in rats fed different pâté formulations. Representative data on tibia length (**A**), wet weight (**B**), moisture (**C**), mineral content (**D**), calcium, phosphorus, and magnesium contents (**E**), and representative tibia images (**F**) from rats from the CP, Si-CP, BP, and Si-BP groups. Data are expressed as mean ± standard deviation (*n* = 7; *n* = 3 for mineral composition). Different letters (a < b < c) indicate statistically significant differences among groups (95% CIs of the mean differences not crossing zero, bootstrap procedure with 1000 resamples, one-way ANOVA, and Tukey’s post-hoc test). CP: commercial pâté. Si-CP: silicon-enriched commercial pâté. BP: biopolymeric pâté. Si-BP: silicon-enriched biopolymeric pâté.

Figure 4 shows tibia length (A), wet weight (B), moisture (C), mineral content (D), and calcium, phosphorus, and magnesium contents (E). Si-CP rats did not show significant differences in tibial parameters compared with CP ones. Nevertheless, tibias from BP rats were slightly shorter, tended to be lighter and showed lower moisture content compared with the CP group. Conversely, in the Si-BP group, tibias were longer and heavier than in the CP and BP groups. No differences were observed in the percentage of tibia mineral content, although Si-BP rats presented higher phosphorus and magnesium content than the rest of the other groups.

### 3.5. Associations of Dietary Fat and Silicon Intake with Metabolic, Hormonal and Bone Parameters

**Figure 5:**
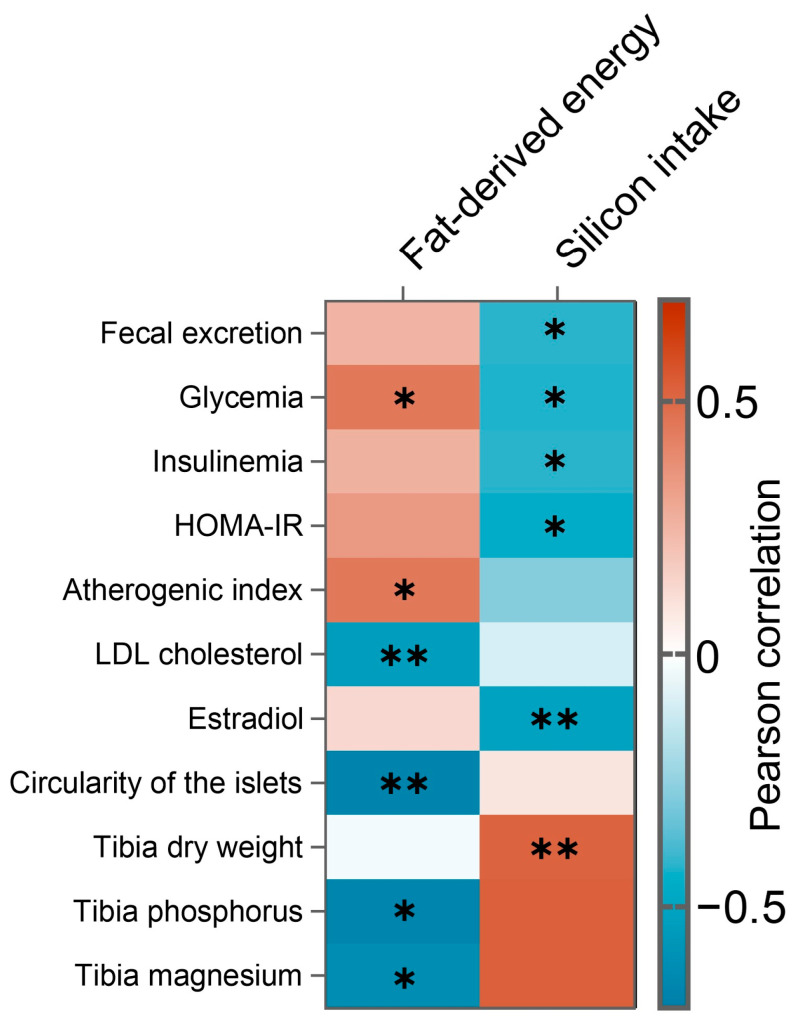
Correlation heatmap representing percentage of fat-derived energy and silicon intake in relation to metabolic, hormonal, and bone parameters. Orange and blue colors represent positive and negative correlations, respectively, according to the color scale. Asterisks indicate statistically significant correlations (* *p* < 0.05; ** *p* < 0.01). Only parameters showing significant associations with fat-derived energy or silicon intake are displayed.

Pearson product–moment correlations were performed to establish the relationships of the percentage of fat-derived energy and silicon intake with fecal excretion, and ponderal, serum, and bone parameters. Figure 5 summarizes all significant correlations, which are highlighted with asterisks. Only those variables which display significant correlation with fat-derived energy or silicon intake are depicted in Figure 5. Fat-derived energy correlated negatively with LDL cholesterol (r = −0.538, *p* = 0.003), islet circularity (r = −0.671, *p* < 0.001), and tibial phosphorus (r = −0.666, *p* < 0.05) and magnesium (r = −0.622, *p* < 0.05), while it correlated positively with serum glucose (r = 0.447, *p* = 0.017) and atherogenic index (r = 0.446, *p* = 0.017). Regarding silicon intake, a positive correlation was found with tibia dry weight (r = 0.512, *p* = 0.005), whereas negative correlations were observed with fecal excretion (r = −0.148, *p* = 0.027), serum glucose (r = −0.424, *p* = 0.024), insulin (r = −0.418, *p* = 0.027), HOMA-IR (r = −0.46, *p* = 0.014), and estradiol (r = −0.516, *p* = 0.005).

## 4. Discussion

In the present study, we propose a non-invasive and feasible nutritional preventive strategy consisting of replacing a processed meat product in the diet, specifically a pâté, with a functional alternative. Such replacement represents a realistic intervention, which takes into consideration the high intake of processed meat in the target population, and can be easily replicated in clinical studies. This study demonstrates that fat reduction and silicon supplementation, alone or in combination, are distinct technological approaches that influenced metabolic outcomes in this diet-induced T2DM female rat model. Even though all experimental groups presented similar daily energy consumption, differences in the lipid profile and energy contribution of each macronutrient, together with silicon supplementation, caused differences in fecal excretion, pancreatic islet morphology, metabolic and hormonal biomarkers, and bone status. Notably, replacing a single food item was sufficient to elicit measurable improvements in the health status of diabetic middle-aged female rats.

Specifically, the nutritional strategy consisted of replacing 3 g of a CP containing 30% of fat with a functional one. The mean energy intake in the CP group was 60.69 kcal/day, with CP providing 9.78 kcal/day, amounting to 16% of the total energy intake. On the other hand, considering a study conducted in 3855 pre-menopausal women, representing the equivalent target population, protein contributed 15% of total energy [20]. Therefore, the protein intake in the present design (12.50% of total energy in the CP group) does not appear to have been overestimated. Furthermore, as the mean daily intake of protein, mainly casein, was 1.95 g, it is important to state that only 0.33 g (approximately 17% of protein intake) was provided by the pâté, which represents a realistic and easily achievable proportion. Three different functional pâtés were formulated. The first one, Si-CP, enriched with silicon as functional ingredient, kept providing 15.76% of daily energy intake, similar to the CP. The second pâté was based on fat reduction through a biopolymeric emulsion as a fat replacer (BP), which provided 10% of the total daily intake within the limits set by the diet quality index, making the diet more balanced. Finally, the third pâté (Si-BP) combined both approaches; the biopolymeric emulsion used as fat replacer was enriched in silicon within the lipid droplets.

Diet-induced T2DM is one of the most widely used models in the literature, since diet is the main factor in the development of metabolic pathologies such as T2DM [21]. In the present study, the HSFHCD provided approximately 60% of total kcal from fat, and from that, 21% of total caloric intake from SFAs. The lipid profile of this diet, with a (PUFAs + MUFAs)/SFAs ratio below 2, indicates a fat quality associated with predisposition to cardiovascular risk [22]. Furthermore, the similar proportions of SFAs and MUFAs resemble the composition found in lard, leading to more pronounced metabolic alterations than those observed with diets containing higher proportions of SFAs or PUFAs, such as those including coconut oil or fish oil [23,24]. In addition, 1.55% cholesterol was incorporated to aggravate diabetic dyslipidemia and to promote the development of metabolic dysfunction-associated steatotic liver disease (MASLD), as previously demonstrated by our research group [12,25,26,27,28]. However, the current literature indicates that females are more resistant to the development of insulin resistance and obesity when subjected to high-fat diets compared to males [29], even when ageing is added as a risk factor. In the present experiment, 1-year-old female rats were fed HSFHCD for 10 weeks, 2 weeks more than previous experiments, because they still exhibited normoglycemia (<100 mg/dL) in the 6th week. After 10 weeks, all these rats developed severe dysmetabolism. Lastly, to avoid the nutritional deficiencies due to vitamin dilution reported when fat is added to a standard diet, we used a commercially available diet specifically adapted to our needs, where carbohydrate content was reduced in favor of fat.

During dietary intervention, CP animals consumed an average of 12.54 g per day, gained 46.43 g of body weight, and excreted 2.09 g dry feces/day. It can be hypothesized that these CP rats presented excessive fecal excretion, compared with a previous experiment analyzing young healthy female rats which reported 1.56 g dry feces/day [13], and with 1-year old males fed a HSFHCD that produced 1.27 g dry feces/day [11]. The suggestion of excessive fecal excretion indicates that CP rats displayed the lowest diet digestibility. Nevertheless, since T2DM is induced by diet, it is unlikely that CP rats would have digested the HSFHCD to a lesser extent from the beginning, given that they showed the most pronounced pathophysiological signs. Thus, although the specific mechanisms involved should be further investigated, disruption of the intestinal barrier in the final stages of the experiment could be linked to the erratic excretion [30]. Another interesting finding in CP rats that supports the discussed hypothesis is the negative silicon digestibility (−44.55%) revealing higher excretion than intake, which implies progressive loss in the body burden of this mineral. This phenomenon has been previously described in aging, inflammation, and atherosclerosis processes [31,32,33], where silicon loss from vascular deposits, particularly from the aorta, has been documented. Mineral deficiencies have been reported in T2DM, but they are usually associated with higher urinary excretion [34], and they are considered an additional risk factor in the prognosis of T2DM [35].

Moreover, CP animals developed the main features of diet-induced T2DM. All rats in this group exhibited fasting hyperglycemia (≥126 mg/dL), and serum insulin reached 20.74 µIU/mL. These values are considered hyperinsulinemic, since several authors indicate that the 9–12 µIU/mL range is the most common in healthy rats [36], although no clear cut-off has been established. The elevated HOMA-IR (mean value of 7.77) further confirms the insulin resistance in this group [37]. These features are expected in T2DM before pancreatic failure, where sustained hyperglycemia triggers compensatory insulin hypersecretion [38]. The CP rats exhibited the lightest pancreases and presented predominantly small islets. Although these were not analyzed further, the small pancreases could have been due to a loss of acinar cell mass [39]. Their mean circularity was 0.816, while islets covered 1.44% of the total area. A potential speculative explanation is that this would appear to be a compensatory mechanism in which the neogenesis of small islets occurs to meet the increased insulin demand required for glucose control [40]. The HSFHCD, including 24% of CP, also triggered hypercholesterolemia (203.7 mg/dL), with cholesterol mainly carried by non-HDL lipoproteins, and triglycerides remaining at normal levels (58.24 mg/dL). The atherogenic index reached −0.67, the least negative, consistent with an increased cardiovascular risk due to T2DM [41].

In the Si-CP group, diatomaceous earth was added as a silicon source directly to lard for pâté preparation. This provided 4 mg of silicon/kg body weight/day, which would have been available throughout the entire digestive tract, being absorbed in the small intestine or exerting its effects from the stomach to the colon. Using the body surface area (BSA) conversion (rat to human, ×0.162) proposed by Reagan-Shaw et al. [42], this corresponds to a human-equivalent dose of 0.65 mg silicon/kg body weight/day, which is approximately 45 mg silicon/day for a 70 kg person. A recent study of Rondanelli et al. [43] proposes a dose of 25 mg silicon/kg body weight/day to obtain beneficial effects in bones. The replacement of organic silicon with diatomaceous earth, as well as the change from male to female rats, led us to evaluate two doses: 2 mg silicon/kg body weight/day and 4 mg silicon/kg body weight/day in healthy female rats [13]. The better results in postprandial triglyceridemia validate the decision to use 4 mg silicon/kg body weight/day in the present study, while they remained below the reported no observed adverse effect level (NOAEL) for oral silica in rats [44]. In the Si-CP group, all parameters related to diet were similar to those in the CP group, except for silicon intake, which was higher by nearly 50%. The Si-CP average diet intake throughout the experiment was 12.79 g/day (62.07 kcal/day), resulting in a weight gain of 59.14 g, comparable to the CP group. Although Si-CP rats did not gain more weight, they displayed the highest diet digestibility as the silicon reduced fecal output in comparison with the CP rats. An inverse correlation was found between silicon intake and fecal excretion. Assuming that CP rats presented exaggerated fecal excretion, Si-CP animals showed values closer to normal. Reinforcement of the intestinal barrier could be a subsequent mechanism explaining this effect, as silicon-enriched meat consumption has previously demonstrated to protect against disruption of intestinal barrier in late-stage T2DM male rats [45,46,47]. The higher intake of silicon and its lower fecal excretion meant that the Si-CP group had the highest silicon digestibility (44.46%), establishing this functional food as a valuable source of bioavailable silicon.

Furthermore, silicon exerted robust protective effects on glycemic metabolism. Si-CP rats developed a more controlled T2DM, whose features included hyperglycemia (146 mg/dL) and mild hyperinsulinemia (12.06 µIU/mL), which was lower than that observed in the CP group. The lower insulin requirement to maintain glucose homeostasis indicated enhanced peripheral insulin sensitivity. Thus, the resulting HOMA-IR reached 4.49, also significantly lower than that exhibited by CP rats. These results are in line with those previously reported by Vidé et al. [48], where silicon-enriched spirulina supplementation also reduced HOMA-IR in male hamsters fed a HSFHCD.

Moreover, although the hypocholesterolemic effect of silicon has been extensively demonstrated in male rats [12,45,46,49,50], total cholesterol in the Si-CP rats (183.1 mg/dL) was similar to that in the CP ones in this experiment. Two key differences may explain the lack of hypocholesterolemic effect. The first is that this study represents the first instance in which silicon intake has been tested in T2DM and hypercholesterolemic female rats, whose response may differ from that of males. The second distinctive feature concerns the mode of delivery of the silicon-supplemented meat product. In previous experiments, organic silicon-enriched meat was incorporated directly into the diet, ensuring simultaneous ingestion with dietary cholesterol. Thereby, silicon could prevent cholesterol absorption, which has been described as one of its most important hypocholesterolemic mechanisms. Here, silicon-enriched pâtes were delivered alone in the morning in an individual cage, separate from the diet, which limited the simultaneous presence of silicon and dietary cholesterol in the intestine, consequently avoiding the opportunity to inhibit cholesterol absorption. In this regard, it can be suggested that Si-CP incorporated into a full meal, alongside other cholesterol-containing foods, could enhance its capacity to reduce serum cholesterol levels. Nevertheless, although total cholesterol did not decrease, Si-CP rats presented significantly lower LDL cholesterol, along with lower levels of triglycerides, both associated with lower cardiovascular risk [51,52]. The underlying mechanism, although it has not been assayed, may be attributed to nutrigenomic changes in the expression of cholesterol and fatty acid transporters in the intestine or liver, leading to both a reduction in lipid uptake and an increase in cholesterol excretion through reverse cholesterol transport and trans-intestinal cholesterol excretion. Another additional mechanism that has been ascribed to silicon hypocholesterolemic effect is the increase in bile excretion [50]. However, considering the significant reduction in fecal excretion registered in Si-CP animals in comparison to the CP group, this hypothesis seems inconsistent with this experiment.

PRP/MTG/κC biopolymeric emulsion with the capacity to reduce lipid digestibility was included as fat replacer in a reformulated pâté (BP), allowing both fat reduction to 15% and partial impairment of fat digestibility. Rats fed a HSFHCD and BP exhibited a mean intake of 12.73 g/day (57.75 kcal/day), similar to CP ones, although the amount of total lipids ingested was significantly lower and their profile was different. Lipid intake and the percentage of daily energy derived from fat was reduced in BP rats compared with their CP counterparts by −8.39% and −3.75%, respectively. Specifically, daily intake of SFAs and MUFAs was reduced by −10.18% and −9.59%, respectively. Furthermore, although BP rats gained the same body weight and exhibited the same serum glucose levels as CP ones, their fasting insulin and HOMA-IR tended to be lower, suggesting better insulin sensitivity. In fact, pancreatic islet morphology confirmed this, as BP rats presented fewer small islets with greater circularity than CP rats, which reflects diminished metabolic stress for the pancreas [39], probably due to a lower demand of insulin secretion. In this sense, correlation heatmap revealed an inverse relationship between fat-derived energy and circularity of the islets, highlighting that the consumption of BP formulation preserved islets because insulin demands did not increase so sharply compared with those groups fed with CP. Regarding serum lipids, a tendency towards lower serum triglyceride levels was observed in BP rats, while cholesterol and lipoprotein-carried cholesterol levels were equivalent to those of CP rats. The lack of significant results suggests that partial fat replacement, despite lowering dietary saturated fat intake, was insufficient to induce short-term changes in lipemia.

For the combined strategy, the PRP/MTG/κC biopolymeric emulsion was enriched in silicon, providing 4 mg of silicon/kg body weight/day. This combination reduced in vitro fat digestion, even more than the biopolymer alone without silicon [15]. However, it should be considered that the silicon was within the lipid droplet covered with the biopolymer, representing a key difference in its bioavailability and biological activity compared with Si-CP. Si-BP rats exhibited a mean daily intake of 12.88 g (58.51 kcal/day), with the same composition as the BP group and significantly reduced in SFAs and MUFAs compared with the CP and Si-CP ones. Silicon intake was equivalent to that of the Si-CP rats, although total fecal excretion and silicon excretion were higher (+23.08 and +66.67%, respectively), resulting in a silicon digestibility of 7.71%, significantly lower than the Si-CP and BP groups. The lower digestibility of silicon was expected due to its delayed release from the lipid droplet, which can provide a higher concentration of silicon in the colonic region, with a different range of effects and systemic consequences. Through the experiment, Si-BP rats gained 364.14 g in weight, without differences from the rest of the groups, although they developed mild T2DM. They presented mild hyperglycemia (129.9 mg/dL), significantly lower than the other groups, and fasting serum insulin (9.49 µIU/mL) even remained in normal range. Consequently, these animals were borderline for insulin sensitive, with a mean HOMA-IR index of 3.02. Their pancreases also exhibited healthier characteristics than those of the CP group due to lower metabolic stress. Si-BP rats presented fewer small islets, more medium-sized ones with greater circularity, and reduced pancreatic area coverage. This improvement was similar to that in the BP group. Regarding serum lipids, Si-BP animals displayed a significantly lower atherogenic index, with higher LDL and HDL cholesterol than their CP, Si-CP, and BP counterparts, and lower triglycerides than the CP group. These results are promising, as increasing HDL cholesterol is considered cardioprotective. There are no previous results describing this effect after silicon supplementation, and the mechanisms involved should be studied more deeply. On the other hand, LDL cholesterol increase could be linked to a faster VLDL transformation, as VLDL tended to decrease in the same proportion, or to a delay in uptake by the liver [53,54]. On top of that, the atherogenic index of Si-BP group was the lowest, bringing to light the impact of this simple intervention. Therefore, the combined strategy provided the greatest improvement in terms of glycemic control and the least deleterious lipid profile, better than silicon inclusion or fat replacement by biopolymeric emulsion alone. The correlation heatmap enabled division of which effects may have been due to fat reformulation, and which are likely to be a consequence of silicon addition. While silicon decreased fecal excretion, serum insulin levels, and HOMA-IR, the specific reformulation with the biopolymeric emulsion, where silicon is incorporated inside lipid droplets, seemed to impact the islets’ morphology and increase the atherogenic index. Both strategies showed a synergistic hypoglycemic effect. These findings underscore the potential of food reformulation strategies, as modifying the accessibility of dietary fat and inclusion of bioactive compounds can mitigate the diabetogenic impact of high-fat diets without necessarily reducing food intake.

Importantly, T2DM development was accompanied by hormonal alterations. The CP rats exhibited the highest concentrations of fasting serum ghrelin, similar to those reported in patients with T2DM, linked with insulin resistance [55]. All nutritional interventions tended to reduce serum ghrelin without affecting food intake, probably reflecting lower insulin resistance in these groups, although ghrelin reduction reached statistical significance only in the Si-CP rats. Similar results were observed by Şengün et al. (2024), who reported that nutritional intervention reduced insulin resistance and ghrelin levels in overweight prediabetic women [56]. In addition to the indirect effect through improving insulin sensitivity, there also appears to be an upstream effect through somatostatin induction, which regulates ghrelin concentrations. Thus, somatostatin downregulates gastric ghrelin secretion and promotes a biphasic effect on gastric emptying. It causes faster early gastric emptying, mainly affecting aqueous components, and slower late gastric emptying, which mainly affects fatty compounds [57]. Hence, concordant findings were observed in healthy rats orally administered with diatomaceous earth as a silicon source, which exhibited faster gastric emptying, but fats remained retained [13]. Moreover, a local direct effect through the gastric mucosa should be considered, which may explain the more pronounced ghrelin reduction observed in the Si-CP group, where silicon was bioavailable, compared with the Si-BP group, where silicon absorption was restricted. Furthermore, hepatic insulin resistance also impedes IGF-1 secretion [58]. Trends towards higher IGF-1 concentrations were found in the Si-CP and BP groups, consistent with lower insulin resistance and improved insulin secretion. Meanwhile, in Si-BP insulin-sensitive animals, lower serum insulin levels also led to lower IGF-1 serum concentrations.

It has been extensively documented that estrogens exert protective roles in females against many metabolic diseases, particularly in relation to cardiovascular and bone health. Nevertheless, estradiol levels, although within the physiological range, were reduced in silicon-supplement groups (Si-CP and Si-BP), as depicted in the correlation heatmap. Regarding this result, which could be incorrectly associated to deleterious effects in silicon groups, two hypotheses can be proposed. According to the first of these, the CP group would display abnormally high estradiol levels as a consequence of hyperinsulinemia [59], and Si-CP or Si-BP consumption would keep them in a more normal range. In the second possibility, silicon would be able to indirectly activate estradiol pathways and consequently, it would exert negative feedback in the axis. Even though the animals were over one year old, they did not show reproductive senescence, confirming that metabolic adaptations occurred under preserved reproductive activity, and all rats were in the diestrus phase. Importantly, estradiol reduction was not associated with worse glycemic control, increased cardiovascular risk, or signs of bone fragility in Si-CP or Si-BP animals. Regarding bone health, Si-BP rats showed greater tibia length and wet weight than their CP and BP counterparts. Moreover, despite similar mineral content in the tibias of all groups, ashes from Si-BP animals exhibited higher phosphorous and magnesium content. Within the physiological range, higher magnesium content is related to osteoblast activation and healthy bones, reducing their potential fragility [60]. Nonetheless, it should be noted that the small sample size may have increased sensitivity to outliers and this limits extrapolation of these results beyond the experimental model. Therefore, these findings should be interpreted within the context of this controlled preclinical setting.

## 5. Conclusions

In conclusion, the replacement of a single potentially atherogenic food item with a metabolically healthier alternative is a realistic nutritional strategy that demonstrated significant improvements in middle-aged female rats with a diet-induced T2DM. Although the three experimental and functional pâtés exhibited efficacy in different parameters, Si-BP consumption yielded the most favorable results. Hence, the reformulation of the pâté with biopolymeric fat replacer and silicon addition in the lard droplets improved energy utilization, alleviated pancreatic stress, improved insulin sensitivity, regulated lipoprotein profiles while maintaining essential hormonal conditions, and showed protective effects on bone health. Despite the lower digestibility of silicon in the Si-BP group compared with Si-CP, these findings are interesting, suggesting the involvement of colonic mechanisms that deserve more research. The present study provides further evidence about the antidiabetic effect of silicon, being the first to evaluate middle-aged female rats. Silicon-enriched pâté could be a suitable nutritional adjuvant therapy for T2DM patients.

## Figures and Tables

**Table 1:** Composition and nutritional values of the different pâté formulations.

	CP	Si-CP	BP	Si-BP
**Ingredients (g/100 g)**				
Meat	19.16	19.16	19.16	19.16
Pork liver	33.00	33.00	33.00	33.00
Lard	27.22	27.22	5.12	5.12
Emulsion	0	0	17.50	17.50
Water	14.80	14.80	19.40	19.40
Additives *	5.82	5.82	5.82	5.82
**Composition (g/100 g)**				
Crude protein	10.84	10.89	11.30	11.44
Total carbohydrates	3.00	2.95	2.54	2.16
Crude fat	30.08	30.16	15.39	15.41
Crude fiber	0	0	0	0
Crude ash	3.66	3.70	4.53	4.88
Humidity	52.42	52.30	66.24	66.11
Cholesterol	0.156	0.156	0.14	0.14
Saturated fatty acids (SFAs)	12.19	12.22	6.16	6.17
Unsaturated fatty acids (UFAs)	15.80	15.85	7.96	7.97
Monounsaturated fatty acids (MUFAs)	12.51	12.55	6.12	6.13
Polyunsaturated fatty acids (PUFAs)	3.29	3.30	1.84	1.84
**Nutritional values**				
% Energy from proteins	13.30	13.33	23.31	23.70
% Energy from lipids	83.02	83.06	71.44	71.83
% Energy from carbohydrates	3.68	3.61	5.24	4.47
Metabolizable energy (kcal/100 g)	326.08	326.8	193.87	193.09

* Additives: 2 g sodium chloride/100 g, 2 g milk powder/100 g, 1 g sodium caseinate/100 g, 0.5 g sodium tripolyphosphate/100 g, and 0.32 g flavoring/100 g. CP: commercial pâté. Si-CP: silicon-enriched commercial pâté. BP: biopolymeric pâté. Si-BP: silicon-enriched biopolymeric pâté.

**Table 2:** Dietary intake, water intake, fecal parameters, and silicon balance in rats fed different pâté formulations.

	CP Group	Si-CP Group	BP Group	Si-BP Group
Mean diet intake (g/day)	12.54 ± 0.31	12.79 ± 0.70	12.73 ± 0.82	12.88 ± 1.06
Proteins (g/day)	1.90 ± 0.05	1.94 ± 0.12	1.95 ± 0.13	1.97 ± 0.18
Carbohydrates (g/day)	3.35 ± 0.11	3.43 ± 0.24	3.40 ± 0.28	3.44 ± 0.36
Lipids (g/day)	4.41 ± 0.12 bc	4.51 ± 0.26 c	4.04 ± 0.30 a	4.10 ± 0.39 ab
Cholesterol (g/day)	0.15 ± 0.01	0.16 ± 0.01	0.16 ± 0.01	0.16 ± 0.02
Mean energy intake (kcal/day)	60.69 ± 1.67	62.07 ± 3.70	57.75 ± 4.33	58.51 ± 5.69
Water intake (mL/day)	20.86 ± 3.63	18.43 ± 2.70	19.00 ± 4.87	17.43 ± 3.74
Fecal excretion (g dry feces/day)	2.09 ± 0.44 c	1.17 ± 0.02 a	1.37 ± 0.11 b	1.44 ± 0.24 b
Fecal moisture (%)	16.58 ± 2.30	18.90 ± 3.60	16.76 ± 0.36	18.81 ± 3.69
Diet apparent digestibility (%)	82.51 ± 2.74 a	92.40 ± 0.83 c	90.95 ± 1.55 b	90.89 ± 0.97 b
Silicon intake (mg/day)	2.96 ± 0.10 a	4.43 ± 0.21 b	2.96 ± 0.27 a	4.44 ± 0.32 b
Silicon excretion (mg/day)	4.29 ± 1.83 b	2.46 ± 0.63 a	2.16 ± 0.10 a	4.10 ± 0.22 b
Silicon digestibility (%)	−44.55 ± 55.41 a	44.46 ± 11.35 d	27.16 ± 2.12 c	7.71 ± 4.23 b

Data are expressed as mean ± standard deviation (*n* = 7). Different letters (a < b < c < d) indicate statistically significant differences among groups (95% CIs of the mean differences not crossing zero, bootstrap procedure with 1000 resamples, one-way ANOVA, and Tukey’s post-hoc test). CP: commercial pâté. Si-CP: silicon-enriched commercial pâté. BP: biopolymeric pâté. Si-BP: silicon-enriched biopolymeric pâté.

**Table 3:** Ponderal parameters and organ weights in rats fed different pâté formulations.

	CP Group	Si-CP Group	BP Group	Si-BP Group
Final body weight (g)	391.4 ± 54.25	377.8 ± 26.78	361.4 ± 52.70	364.1 ± 13.58
Body weight gain (g)	46.43 ± 23.17 ab	59.14 ± 18.57 b	41.50 ±17.42 a	36.64 ± 13.80 a
Relative gonadal fat weight	0.04 ± 0.006	0.04 ± 0.002	0.04 ± 0.006	0.04 ± 0.006
Relative retroperitoneal fat weight	0.03 ± 0.006	0.02 ± 0.003	0.02 ± 0.003	0.02 ± 0.003
Relative subcutaneous fat weight	0.01 ± 0.003	0.01 ± 0.003	0.01 ± 0.002	0.01 ± 0.001
Relative liver weight	0.05 ± 0.002	0.05 ± 0.003	0.05 ± 0.004	0.05 ± 0.005
Relative pancreas weight	0.003 ± 0.001 a	0.004 ± 0.001 b	0.004 ± 0.001 ab	0.004 ± 0.001 ab

Data are expressed as mean ± standard deviation (*n* = 7). Different letters (a < b) indicate statistically significant differences among groups (95% CIs of the mean differences not crossing zero, bootstrap procedure with 1000 resamples, one-way ANOVA, and Tukey’s post-hoc test). CP: commercial pâté. Si-CP: silicon-enriched commercial pâté. BP: biopolymeric pâté. Si-BP: silicon-enriched biopolymeric pâté.

**Table 4:** Serum biochemical and hormonal parameters of rats fed different pâté formulations.

	CP Group	Si-CP Group	BP Group	Si-BP Group
Initial serum glucose (mg/dL)	92.86 ± 10.48	81.29 ± 12.76	90.71 ± 17.04	86.00 ± 12.88
Intermediate serum glucose (mg/dL)	96.14 ± 10.85	92.71 ± 10.86	90.14 ± 9.69	91.43 ± 5.68
Final serum glucose (mg/dL)	154.9 ± 15.76 b	146.0 ± 11.63 b	145.8 ± 11.83 b	129.1 ± 10.40 a
Insulin (µIU/mL)	20.74 ± 7.44 b	12.06 ± 7.89 a	14.97 ± 7.89 ab	9.49 ± 6.06 a
HOMA-IR	7.77 ± 2.26 b	4.49 ± 3.24 a	5.31 ± 2.66 ab	3.02 ± 1.86 a
Triglycerides (mg/dL)	58.24 ± 9.97 b	47.51 ± 4.46 a	51.63 ± 7.06 ab	46.91 ± 5.13 a
Total cholesterol (mg/dL)	203.7 ± 26.31	183.1 ± 24.16	194.6 ± 34.56	195.2 ± 9.39
VLDL cholesterol (mg/dL)	48.63 ± 11.05 ab	47.66 ± 6.46 b	45.84 ± 14.57 ab	41.36 ± 4.97 a
IDL cholesterol (mg/dL)	60.70 ± 20.83	54.34 ± 13.60	53.10 ± 19.46	47.34 ± 9.19
LDL cholesterol (mg/dL)	42.20 ± 4.98 b	34.63 ± 5.14 a	43.38 ± 5.56 b	48.51 ± 3.26 c
HDL cholesterol (mg/dL)	52.14 ± 5.26 a	46.51 ± 9.55 a	52.23 ± 5.02 a	57.96 ± 4.44 b
Atherogenic index	−0.67 ± 0.08 a	−0.70 ± 0.09 a	−0.73 ± 0.07 a	−0.81 ± 0.05 b
Ghrelin (pg/mL)	78.35 ± 22.85 b	32.49 ± 24.66 a	61.10 ± 48.03 ab	57.09 ± 35.06 ab
IGF-1 (ng/mL)	156.4 ± 86.24 ab	204.6 ± 63.61 b	192.9 ± 55.61 b	110.7 ± 39.24 a
Estradiol (ng/mL)	634.4 ± 98.87 b	555.1 ± 59.86 ab	618.7 ± 65.85 b	528.2 ± 45.00 a

Data are expressed as mean ± standard deviation (*n* = 7). Different letters (a < b < c) indicate statistically significant differences among groups (95% CIs of the mean differences not crossing zero, bootstrap procedure with 1000 resamples, one-way ANOVA, and Tukey’s post-hoc test). CP: commercial pâté. Si-CP: silicon-enriched commercial pâté. BP: biopolymeric pâté. Si-BP: silicon-enriched biopolymeric pâté. HOMA-IR: Homeostatic Model Assessment of Insulin Resistance; VLDL: very low-density lipoprotein; IDL: intermediate-density lipoprotein; LDL: low-density lipoprotein; HDL: high-density lipoprotein; Atherogenic index = log_10_(Triglycerides/HDL cholesterol); IGF-1: insulin-like growth factor 1.

## Data Availability

The original contributions presented in this study are included in the article/Supplementary Materials. Further inquiries can be directed to the corresponding authors.

## Supplementary Materials

The following supporting information can be downloaded at: https://www.mdpi.com/article/10.3390/foods15050878/s1, Figure S1: Representative images of vaginal smears; Figure S2: Representative images of daily fecal excretion; Table S1: Composition of the high-saturated fat high-cholesterol diet (U8958P version 370 purchased from Safe), which was provided to all experimental groups, compared to AIN-93M control diet; Table S2: Statistical results.

## Abbreviations

The following abbreviations are used in this manuscript:

BP	Reduced-fat biopolymeric pâté
CP	Commercial pâté
DE	Diatomaceous earth
ELISA	Enzyme-linked immunosorbent assay
HDL	High-density lipoprotein
HOMA-IR	Homeostatic Model Assessment of Insulin Resistance
HSFHCD	High-saturated-fat high-cholesterol diet
ICP-OES	Inductively coupled plasma optical emission spectrometry
IDL	Intermediate-density lipoprotein
IGF-1	Insulin-like growth factor 1
κC	κ-carrageenan
LDL	Low-density lipoprotein
MASLD	Metabolic dysfunction-associated steatotic liver disease
MTG	Microbial transglutaminase
MUFAs	Monounsaturated fatty acids
NOAEL	No observed adverse effect level
O/W	Oil-in-water emulsion
PRP	Pork rind protein extract
PUFAs	Polyunsaturated fatty acids
SFAs	Saturated fatty acids
Si-BP	Silicon-enriched reduced-fat biopolymeric pâté
Si-CP	Silicon-enriched commercial pâté
T2DM	Type 2 diabetes mellitus
VLDL	Very low-density lipoprotein
