# Supplementary material for: Antidiabetic Effect of Substituting Processed Meat with Reduced-Fat and Diatomaceous Earth-Enriched Pâtés in Middle-Aged Female Wistar Rats
Source: Foods. 2026 Mar 4;15(5):878. doi: 10.3390/foods15050878 (PMC12984183; doi:10.3390/foods15050878)
Supplement: Supplementary file 1 [file foods-15-00878-s001.zip › foods-4120224-supplementary.pdf]

SUPPLEMENTARY TABLES

Table S1. Composition of the high-saturated fat high-cholesterol diet (U8958P version 370 purchased from Safe), which was provided to all experimental groups, compared to AIN-93M control diet.

	**U8958P v370**	**AIN-93M**
**Ingredients**	**g/100g**	
Sucrose	5.25	10
Pregelatinized cornstarch	19.24	46.57
Maltodextrin	7	15.50
Casein	18.9	14
Soybean oil	5	4
Lard	30	0
Cholesterol	1.6	0
Cholic acid	0.32	0
Crude cellulose	5	5
Pre-mixture of vitamins PV AIN 93M_G 1%	1.3	1
Pre-mixture of minerals PM AIN 93M_G 3.5%	4.54	3.5
Sodium bicarbonate	1.05	0
Potassium citrate	0.4	0
Choline bitartrate	0.2	0.25
Dicalcium phosphate	0	0
L-cystine	0.2	0.18
**Composition**	**g/100g**	
Sucrose	7.09	11
Total sugars	7.97	13.03
Starch	23.29	55.14
Nitrogen-free extract	34.13	69.22
Crude protein	16.49	12.49
Crude fat	36.81	4.4
Crude fiber	3.53	3.6
Crude ash	4.15	2.6
Humidity	4.87	7.7
Cholesterol	1.55	0
Active ingredient	0.31	
Calcium (Ca)	0.64	0.496

Phosphorus (P)	0.32	0.251
Sodium (Na)	0.44	0.15
Potassium (K)	0.63	0.369
Medium-chain fatty acids (MCFA)	0.03	
Long-chain fatty acids (LCFA)	33.03	
Saturated fatty acids (SFAs)	12.36	0.59
Unsaturated fatty acids (UFAs)	20.70	3.188
Monounsaturated fatty acids (MUFAs)	14.06	0.764
Polyunsaturated fatty acids (PUFAs)	6.65	2.424
Total omega 3 fatty acids	0.69	0.308
Total omega 6 fatty acids	5.96	2.116
Total omega 7 fatty acids	0.92	
Total omega 9 fatty acids	12.99	
C16:0 Hexadecanoic acid (Palmitic acid)	7.76	0.424
C18:0 Octadecanoic acid (Stearic acid)	4.14	0.146
C16:1 (n-7) cis-Hexadec-9-enoic acid (Palmitoleic acid)	0.92	0.02
C18:1 (n-9) cis-Octadec-9-enoic acid (Oleic acid)	12.99	0.744
C18:2 (n-6) all-cis-9.12-octadecadienoic acid (Linoleic acid)	5.45	2.116
C18:3 (n-3) all-cis-9.12.15-octadecatrienoic acid (Alpha-linolenic acid)	0.69	0.308
	**IU/100g**	
Vitamin A	608.34	470.7
Vitamin D3	162.50	125
Vitamin E (dl-alpha-tocopherol acetate)	11.49	8.3
**Nutritional values**		
% Energy from sugar	5.98	
% Energy from starch	17.45	
% Energy from nitrogen-free extract	25.58	75.6
% Energy from proteins	12.36	13.6
% Energy from lipids	62.06	10.7
Metabolizable energy (kcal/100g)	533.84	366.06

Table S2. Statistical results

Group 1: CP; Group 2: Si-CP; Group 3: BP; Group 4: Si-BP

Mean diet intake (g/day)

	Groups comparison	Mean difference	95% CI_lower bound	95% CI_upper bound
1	2-1	0.26207708	-0.32727	0.8324909
2	3-1	0.18635180	-0.3866384	0.8975766
3	4-1	0.34547258	-0.5408899	1.2103727
4	3-2	-0.07501070	-0.8188854	0.7890427
5	4-2	0.08416268	-0.9457653	1.0216032
6	4-3	0.15917338	-0.8717128	1.1281781

Proteins (g/day)

	Groups comparison	Mean difference	95% CI_lower bound	95% CI_upper bound
1	2-1	0.045569772	-0.05162432	0.1399944
2	3-1	0.047738148	-0.04444444	0.1619492
3	4-1	0.073513036	-0.07499395	0.2193609
4	3-2	0.002283479	-0.11997177	0.1424788
5	4-2	0.028100565	-0.14384725	0.1855499
6	4-3	0.025817086	-0.14399365	0.1866667

Carbohydrates (g/day)

	Groups comparison	Mean difference	95% CI_lower bound	95% CI_upper bound
1	2-1	0.088362953	-0.1126624	0.2819809
2	3-1	0.048290287	-0.1466243	0.2898185
3	4-1	0.091440401	-0.2080928	0.3863108
4	3-2	-0.039823036	-0.2915160	0.2516606
5	4-2	0.003305512	-0.3465545	0.3216243
6	4-3	0.043128548	-0.3082063	0.3710906

Lipids (g/day)

	Groups comparison	Mean difference	95% CI_lower bound	95% CI_upper bound
1	2-1	0.10244863	-0.1150000	0.31249093
2	3-1	-0.36896144	-0.5788607	-0.10818630
3	4-1	-0.30946189	-0.6361856	0.01089577
4	3-2	-0.47114322	-0.7444268	-0.15583333
5	4-2	-0.41250271	-0.7915517	-0.06701270
6	4-3	0.05864051	-0.3408243	0.41498589

Cholesterol (g/day)

	Groups comparison	Mean difference	95% CI_lower bound	95% CI_upper bound
1	2-1	0.005840732	-0.002222222	0.013750000
2	3-1	0.002795451	-0.004444444	0.011552330
3	4-1	0.004347744	-0.009000000	0.017500000
4	3-2	-0.003035082	-0.012500000	0.007141537
5	4-2	-0.001482162	-0.017126525	0.012216577
6	4-3	0.001552920	-0.013333333	0.015000000

Mean energy intake (kcal/day)

	Groups comparison	Mean difference	95% CI_lower bound	95% CI_upper bound
1	2-1	1.4231523	-1.717822	4.4599407
2	3-1	-2.9669825	-6.018607	0.8274256
3	4-1	-2.1471211	-6.894341	2.4699395
4	3-2	-4.3862763	-8.339966	0.2066032
5	4-2	-3.5741112	-9.066174	1.4274955
6	4-3	0.8121651	-4.983260	5.9733333

Water intake (mL/day)

	Groups comparison	Mean difference	95% CI_lower bound	95% CI_upper bound
1	2-1	-2.4426133	-5.833278	0.7222222
2	3-1	-1.8093233	-6.381645	2.5406081
3	4-1	-3.4449718	-7.499546	0.2662432
4	3-2	0.6350047	-3.500000	4.5826276
5	4-2	-1.0022284	-4.629946	2.5974595
6	4-3	-1.6372330	-6.399819	3.0821236

Fecal excretion (g dry feces/day)

	Groups comparison	Mean difference	95% CI_lower bound	95% CI_upper bound
1	2-1	-0.9247458	-1.26964973	-0.6628967
2	3-1	-0.7201150	-1.07594538	-0.4317706
3	4-1	-0.6453917	-1.06416169	-0.3093446
4	3-2	0.2035447	0.12813170	0.2790848
5	4-2	0.2779013	0.08557085	0.4755975
6	4-3	0.0743566	-0.12938250	0.2821194

Fecal moisture (%)

	Groups comparison	Mean difference	95% CI_lower bound	95% CI_upper bound
1	2-1	2.28403457	-1.1258553	5.3947375
2	3-1	0.20432678	-1.7105499	2.0433741
3	4-1	2.23428742	-1.3163333	5.5880213
4	3-2	-2.07714749	-4.7280901	0.7170841
5	4-2	-0.04956988	-3.8182271	3.9195021
6	4-3	2.02757762	-0.8630556	4.8186616

Diet digestibility (%)

	Groups comparison	Mean difference	95% CI_lower bound	95% CI_upper bound
1	2-1	9.90578356	7.756523	11.90038288
2	3-1	8.44774846	6.034519	10.62931291
3	4-1	8.37875268	6.178189	10.43982274
4	3-2	-1.44786831	-2.741570	-0.05511573
5	4-2	-1.50964815	-2.487447	-0.58847094
6	4-3	-0.06177984	-1.369695	1.22437939

Silicon intake (mg/day)

	Comparison	Mean difference	95% CI_lower bound	95% CI_upper bound
1	2-1	1.470673849	1.2866667	1.6428571
2	3-1	-0.001367224	-0.1888889	0.2313076
3	4-1	1.484113223	1.2190718	1.7500000
4	3-2	-1.471839473	-1.7111111	-1.1944309
5	4-2	0.013567787	-0.2998185	0.3000000
6	4-3	1.485407260	1.1571429	1.7897459

Silicon excretion (mg/day)

	Comparison	Mean difference	95% CI_lower bound	95% CI_upper bound
1	2-1	-1.8238903	-3.3142403	-0.4875665
2	3-1	-2.1295251	-3.6243649	-0.8801270
3	4-1	-0.1867070	-1.6616532	1.0829099
4	3-2	-0.3098705	-0.7799435	0.1833333
5	4-2	1.6323103	1.1270245	2.1165820
6	4-3	1.9421807	1.7667876	2.1305556

Silicon digestibility (%)

	Comparison	Mean difference	95% CI_lower bound	95% CI_upper bound
1	2-1	88.91349	50.07828	134.014909
2	3-1	71.72225	33.95229	116.834129
3	4-1	52.26000	13.38801	96.856353

4	3-2	-17.06632	-26.11697	-8.261079
5	4-2	-36.48876	-45.70188	-28.103909
6	4-3	-19.42244	-22.82888	-15.538105

SFAs (g/day)

	Comparison	Mean difference	95% CI_lower bound	95% CI_upper bound
1	2-1	0.03648957	-0.0350000	0.10598614
2	3-1	-0.15632683	-0.2255556	-0.07006775
3	4-1	-0.13219257	-0.2418083	-0.02516743
4	3-2	-0.19273203	-0.2822081	-0.09000000
5	4-2	-0.16894760	-0.2938627	-0.05447550
6	4-3	0.02378443	-0.1084135	0.14220811

MUFAs (g/day)

	Comparison	Mean difference	95% CI_lower bound	95% CI_upper bound
1	2-1	0.03798872	-0.04433333	0.11749093
2	3-1	-0.16503247	-0.24598064	-0.06405262
3	4-1	-0.14209349	-0.26931398	-0.01788187
4	3-2	-0.20292324	-0.30707865	-0.08169691
5	4-2	-0.18036066	-0.32574506	-0.04670901
6	4-3	0.02256258	-0.12989415	0.16109058

PUFAs (g/day)

	Comparison	Mean difference	95% CI_lower bound	95% CI_upper bound
1	2-1	0.01759322	-0.02100000	0.05499153
2	3-1	-0.03033654	-0.06777778	0.01598367
3	4-1	-0.01684491	-0.07465082	0.04000000
4	3-2	-0.04788557	-0.09665699	0.00856538
5	4-2	-0.03447308	-0.10149819	0.02700000
6	4-3	0.01341249	-0.05742590	0.07795843

Proteins (%)

	Comparison	Mean difference	95% CI_lower bound	95% CI_upper bound
1	2-1	0.00514734	0.00000000	0.008333333
2	3-1	0.95481733	0.89333333	1.000000000
3	4-1	0.98143668	0.89400000	1.069915316
4	3-2	0.94955759	0.88293275	0.997500000
5	4-2	0.97813188	0.89056489	1.065000000
6	4-3	0.02857430	-0.07104054	0.136277389

Lipids (%)

	Comparison	Mean difference	95% CI_lower bound	95% CI_upper bound
1	2-1	-0.05456317	-0.2185513	0.1192868
2	3-1	-2.43245462	-2.5150000	-2.3446429
3	4-1	-2.40662779	-2.5040000	-2.3010191
4	3-2	-2.37793491	-2.5487901	-2.2225454
5	4-2	-2.35688855	-2.5199915	-2.1800635
6	4-3	0.02104636	-0.0600000	0.1154026

Carbohydrates (%)

	Comparison	Mean difference	95% CI_lower bound	95% CI_upper bound
1	2-1	0.05516349	-0.1259111	0.2275000
2	3-1	1.47046498	1.3555556	1.6114286
3	4-1	1.42804824	1.2412348	1.6022109
4	3-2	1.41545822	1.2300423	1.6266667
5	4-2	1.37585360	1.1334558	1.5939570
6	4-3	-0.03960462	-0.2398730	0.1499153

Final body weight (g)

	Comparison	Mean difference	95% CI_lower bound	95% CI_upper bound
1	2-1	-13.762991	-56.44201	31.580309
2	3-1	-31.821274	-85.92850	26.230946
3	4-1	-27.911313	-69.77809	13.409913
4	3-2	-18.129491	-61.43344	23.827405
5	4-2	-14.284823	-37.90154	9.566227
6	4-3	3.844668	-34.69098	42.526559

Body weight gain (g)

	Comparison	Mean difference	95% CI_lower bound	95% CI_upper bound
1	2-1	12.798273	-10.69003	36.295766
2	3-1	-5.882294	-27.76684	16.011602
3	4-1	-10.236033	-29.43718	10.874546
4	3-2	-18.668754	-38.47158	-1.403952
5	4-2	-23.038883	-40.37182	-5.001482
6	4-3	-4.370129	-19.99905	13.299234

Relative gonadal fat weight

	Comparison	Mean difference	95% CI_lower bound	95% CI_upper bound
1	2-1	-0.002211872	-0.0069294072	0.002773941
2	3-1	-0.003934054	-0.0102730484	0.002476363
3	4-1	0.002093578	-0.0043828623	0.008512571
4	3-2	-0.001730184	-0.0061118133	0.003049577
5	4-2	0.004291415	-0.0007592378	0.009056853
6	4-3	0.006021599	-0.0002569614	0.011978390

Relative retroperitoneal fat weight

	Comparison	Mean difference	95% CI_lower bound	95% CI_upper bound
1	2-1	-0.0025662989	-0.007570622	0.0025821014
2	3-1	-0.0032767406	-0.008462436	0.0019446734
3	4-1	-0.0038803644	-0.008901683	0.0009165608
4	3-2	-0.0007179300	-0.004284430	0.0024143366
5	4-2	-0.0013303006	-0.005195569	0.0019819546
6	4-3	-0.0006123706	-0.003733091	0.0023841108

Relative subcutaneous fat weight

	Comparison	Mean difference	95% CI_lower bound	95% CI_upper bound
1	2-1	-0.0029121942	-0.006277072	9.041419e-05
2	3-1	-0.0021456728	-0.005123941	5.000000e-04
3	4-1	-0.0015950142	-0.004060427	7.848070e-04
4	3-2	0.0007658384	-0.001857143	3.374546e-03
5	4-2	0.0013132499	-0.001190174	3.791515e-03
6	4-3	0.0005474115	-0.001450000	2.285513e-03

Relative liver weight

	Comparison	Mean difference	95% CI_lower bound	95% CI_upper bound
1	2-1	0.0018201798	-0.0008191924	0.004247883
2	3-1	0.0027232914	-0.0000824865	0.005840717
3	4-1	0.0020311378	-0.0026652553	0.005444041
4	3-2	0.0009041240	-0.0023663844	0.004427210
5	4-2	0.0002141152	-0.0044437387	0.004099577
6	4-3	-0.0006900088	-0.0054985886	0.003398730

Relative pancreas weight

	Comparison	Mean difference	95% CI_lower bound	95% CI_upper bound
1	2-1	7.251309e-04	1.066341e-04	0.0013175258
2	3-1	3.064011e-04	-1.168832e-04	0.0007500000
3	4-1	6.555410e-04	-1.541673e-05	0.0013400277
4	3-2	-4.179909e-04	-1.098776e-03	0.0002216388
5	4-2	-6.901497e-05	-8.488940e-04	0.0008337182
6	4-3	3.489760e-04	-4.050000e-04	0.0011474616

Mean islet area

	Comparison	Mean difference	95% CI_lower bound	95% CI_upper bound
1	2-1	-1190.38508	-4469.504	1915.620
2	3-1	-904.11039	-4294.830	2248.836
3	4-1	-1174.52370	-4583.288	2016.107
4	3-2	286.38197	-1490.188	1822.322
5	4-2	16.37229	-1613.703	1554.480
6	4-3	-270.00967	-1653.201	1194.525

Percentage of islets tertile 1

	Comparison	Mean difference	95% CI_lower bound	95% CI_upper bound
1	2-1	-4.0865102	-23.94710	13.142510
2	3-1	-16.2986591	-31.32350	-2.302743

	Comparison	Mean difference	95% CI_lower bound	95% CI_upper bound
3	4-1	-16.7590418	-32.43047	-1.403319
4	3-2	-12.2300235	-27.54509	4.494165
5	4-2	-12.7220250	-28.22949	4.103299
6	4-3	-0.4920014	-12.00546	11.888750

Percentage of islets tertile 2

	Comparison	Mean difference	95% CI_lower bound	95% CI_upper bound
1	2-1	1.8885654	-9.5824342	14.246748
2	3-1	8.6685222	-1.8780081	19.499545
3	4-1	8.3960669	0.3061054	18.304462
4	3-2	6.7777160	-6.8326162	19.349479
5	4-2	6.5234245	-4.0558241	17.193582
6	4-3	-0.2542915	-9.9913240	9.394729

Percentage of islets tertile 3

	Comparison	Mean difference	95% CI_lower bound	95% CI_upper bound
1	2-1	2.1978568	-16.453841	21.68219
2	3-1	7.6343934	-9.134058	23.99712
3	4-1	8.3658689	-8.652875	24.34288
4	3-2	5.4566445	-9.661531	19.60139
5	4-2	6.2015803	-7.268011	20.46981
6	4-3	0.7449358	-9.479555	13.17934

Area occupied by islets (%)

	Comparison	Mean difference	95% CI_lower bound	95% CI_upper bound
1	2-1	-0.49020236	-0.9159964	-0.1131690
2	3-1	-0.55751059	-0.9988607	-0.1156081
3	4-1	-0.61352324	-1.0569687	-0.1884455
4	3-2	-0.06866970	-0.4688603	0.3259081
5	4-2	-0.12564677	-0.4882910	0.2548403
6	4-3	-0.05697707	-0.5088445	0.4099153

Islet circularity

	Comparison	Mean difference	95% CI_lower bound	95% CI_upper bound
1	2-1	0.01875583	-0.014795721	0.04410442
2	3-1	0.05296753	0.041428571	0.06499365
3	4-1	0.06395132	0.050367123	0.07699365
4	3-2	0.03421896	0.008888889	0.06997177
5	4-2	0.04531727	0.018217310	0.07968214
6	4-3	0.01109831	-0.004166667	0.02532486

Initial glycemia (mg/dL)

	Comparison	Mean difference	95% CI_lower bound	95% CI_upper bound
1	2-1	-11.437835	-23.805895	1.465699
2	3-1	-2.105627	-15.663844	12.965336
3	4-1	-6.972255	-18.909257	5.886662
4	3-2	9.358107	-5.277072	25.648160
5	4-2	4.495130	-9.500000	18.264533
6	4-3	-4.862977	-20.990120	11.038068

Intermediate glycemia (mg/dL)

	Comparison	Mean difference	95% CI_lower bound	95% CI_upper bound
1	2-1	-3.465006	-14.698095	8.221113
2	3-1	-6.090539	-17.096014	5.333212
3	4-1	-4.647702	-13.373095	4.774955
4	3-2	-2.657224	-13.428481	7.854421
5	4-2	-1.220193	-10.271779	7.868014
6	4-3	1.437031	-6.924728	10.092819

Final glycemia (mg/dL)

	Comparison	Mean difference	95% CI_lower bound	95% CI_upper bound

	Comparison	Mean difference	95% CI_lower bound	95% CI_upper bound
1	2-1	-9.2750085	-23.83984	5.017060
2	3-1	-9.4663012	-24.32245	4.875105
3	4-1	-26.1713586	-40.60289	-12.756255
4	3-2	-0.1751405	-12.66932	11.432039
5	4-2	-16.9146205	-29.38737	-6.037151
6	4-3	-16.7394801	-29.00754	-6.602168

Insulinemia (µIU/mL)

	Comparison	Mean difference	95% CI_lower bound	95% CI_upper bound
1	2-1	-8.780272	-17.378412	-0.7774864
2	3-1	-5.891506	-14.323410	2.0272070
3	4-1	-11.223342	-18.967688	-4.1178506
4	3-2	2.874586	-6.086161	10.6518670
5	4-2	-2.474266	-10.555459	4.9335344
6	4-3	-5.348852	-12.559891	1.9886874

HOMA-IR

	Comparison	Mean difference	95% CI_lower bound	95% CI_upper bound
1	2-1	-3.3342916	-6.187486	-0.1795701
2	3-1	-2.5168082	-5.074881	0.1648822
3	4-1	-4.7548297	-7.140553	-2.5683804
4	3-2	0.8126518	-2.604005	3.7338755
5	4-2	-1.4327303	-4.506031	1.3112683
6	4-3	-2.2453821	-4.610258	0.1181428

Triglyceridemia (mg/dL)

	Comparison	Mean difference	95% CI_lower bound	95% CI_upper bound
1	2-1	-10.6498258	-18.430319	-1.926019
2	3-1	-6.5197240	-14.878408	2.754459
3	4-1	-11.2859773	-19.320688	-2.436735
4	3-2	4.1237230	-1.956103	10.159550
5	4-2	-0.6529208	-5.786316	4.123573
6	4-3	-4.7766438	-11.158889	1.460836

Cholesterolemia (mg/dL)

	Comparison	Mean difference	95% CI_lower bound	95% CI_upper bound
1	2-1	-21.2717975	-47.459763	6.531421
2	3-1	-9.3063490	-39.887140	21.496354
3	4-1	-8.7819602	-28.603423	12.082698
4	3-2	11.9547914	-18.781708	39.881763
5	4-2	12.4689906	-6.546649	31.902032
6	4-3	0.5141992	-22.660080	26.263401

VLDL cholesterol (mg/dL)

	Comparison	Mean difference	95% CI_lower bound	95% CI_upper bound
1	2-1	-0.9959596	-10.18303	9.2319885
2	3-1	-2.6236579	-15.71786	10.5312072
3	4-1	-7.3213272	-16.20362	2.2364345
4	3-2	-1.6013643	-14.07356	8.6247690
5	4-2	-6.3061665	-12.66355	-0.9208493
6	4-3	-4.7048022	-14.18447	7.0972551

IDL cholesterol (mg/dL)

	Comparison	Mean difference	95% CI_lower bound	95% CI_upper bound
1	2-1	-6.638083	-23.75605	11.317413
2	3-1	-7.901275	-28.06053	13.128332
3	4-1	-13.443104	-28.61025	4.169982
4	3-2	-1.261520	-19.78638	14.979740
5	4-2	-6.824515	-19.71272	4.757500
6	4-3	-5.562996	-20.16370	11.079395

LDL cholesterol (mg/dL)

	Comparison	Mean difference	95% CI_lower bound	95% CI_upper bound
1	2-1	-7.7745154	-12.9303537	-2.196207
2	3-1	0.9978433	-4.7588609	6.154297
3	4-1	6.1843472	1.5609613	10.455696
4	3-2	8.7497642	3.3149095	13.456649
5	4-2	13.9467428	9.4585154	18.581075

6	4-3	5.1969786	0.9370651	10.275512

HDL cholesterol (mg/dL)

	Comparison	Mean difference	95% CI_lower bound	95% CI_upper bound
1	2-1	-5.8646648	-13.3825128	3.101545
2	3-1	0.2192869	-4.9597752	5.477458
3	4-1	5.7937297	0.4912526	10.869127
4	3-2	6.0678941	-2.4017548	13.336210
5	4-2	11.6499672	3.4782277	18.535839
6	4-3	5.5820731	0.5787932	10.579402

Atherogenic index

	Comparison	Mean difference	95% CI_lower bound	95% CI_upper bound
1	2-1	-0.02653803	-0.1091109	0.05783333
2	3-1	-0.05128616	-0.1228559	0.02217764
3	4-1	-0.13669415	-0.2005759	-0.06935823
4	3-2	-0.02466058	-0.1108863	0.05268927
5	4-2	-0.11022450	-0.1820261	-0.03845130
6	4-3	-0.08556392	-0.1378396	-0.02290381

Ghrelin (pg/mL)

	Comparison	Mean difference	95% CI_lower bound	95% CI_upper bound
1	2-1	-45.046148	-68.77193	-18.133295
2	3-1	-17.361159	-55.17827	24.384956
3	4-1	-20.905154	-51.16016	8.303416
4	3-2	27.658761	-10.46710	66.478553
5	4-2	24.132116	-7.10016	55.458042
6	4-3	-3.526645	-47.61820	39.953006

IGF-1 (ng/mL)

	Comparison	Mean difference	95% CI_lower bound	95% CI_upper bound
1	2-1	49.97331	-32.42634	126.65977
2	3-1	34.55128	-47.19039	102.60553
3	4-1	-45.43974	-113.32178	21.56041
4	3-2	-15.19319	-76.72708	48.13961
5	4-2	-95.20796	-148.74124	-39.35692
6	4-3	-80.01476	-131.52380	-30.85749

Estradiol (ng/mL)

	Comparison	Mean difference	95% CI_lower bound	95% CI_upper bound
1	2-1	-80.54452	-164.542738	4.15387
2	3-1	-16.93811	-101.840938	62.70947
3	4-1	-106.20182	-191.957557	-26.77284
4	3-2	63.49617	-1.215102	129.62318
5	4-2	-25.79213	-80.035716	29.44252
6	4-3	-89.28830	-148.441091	-33.46537

Tibia length

	Comparison	Mean difference	95% CI_lower bound	95% CI_upper bound
1	2-1	-0.06808358	-0.21249038	0.07423329
2	3-1	-0.15193244	-0.27833333	-0.04375866
3	4-1	0.13561399	0.02916667	0.25000000
4	3-2	-0.08459085	-0.26663587	0.07389838
5	4-2	0.20294472	0.03573078	0.35453583
6	4-3	0.28753557	0.15750706	0.42699153

Tibia wet weight

	Comparison	Mean difference	95% CI_lower bound	95% CI_upper bound
1	2-1	0.03050117	-0.054838089	0.114272104
2	3-1	-0.03813534	-0.113325635	0.035000000
3	4-1	0.08885501	0.005409108	0.165696139
4	3-2	-0.06880290	-0.133325772	-0.007274267
5	4-2	0.05816327	-0.017282085	0.123330309
6	4-3	0.12696617	0.063354504	0.194556911

Tibia moisture (%)

	Comparison	Mean difference	95% CI_lower bound	95% CI_upper bound
1	2-1	-1.1680600	-2.8754924	0.6686584
2	3-1	-1.6687976	-2.8510440	-0.4576606
3	4-1	0.5926542	-1.3891808	2.2122358
4	3-2	-0.5042349	-2.1241350	0.9874213
5	4-2	1.7571931	-0.4049274	3.6719873
6	4-3	2.2614281	0.4056767	3.7000000

Tibia mineral content (%)

	Comparison	Mean difference	95% CI_lower bound	95% CI_upper bound
1	2-1	2.63899935	-1.143811381	6.6174255
2	3-1	2.90376732	-0.007041909	6.2984327
3	4-1	-0.08633328	-4.426643571	4.7120403
4	3-2	0.27056053	-2.317427414	3.0587725
5	4-2	-2.71951550	-6.494795277	1.6435032
6	4-3	-2.99007603	-6.095428019	0.8168716

Tibia calcium

	Comparison	Mean difference	95% CI_lower bound	95% CI_upper bound
1	2-1	1.1207789	-1.3000000	4.500000
2	3-1	1.9556828	-0.9250000	5.944919
3	4-1	2.3785082	-0.0500000	6.060000
4	3-2	0.8724309	-0.6500000	2.632486
5	4-2	1.5795699	0.3019054	3.187714
6	4-3	0.7242377	-0.7994919	2.350000

Tibia phosphorus

	Comparison	Mean difference	95% CI_lower bound	95% CI_upper bound
1	2-1	0.3855022	-0.6000000	1.700000
2	3-1	0.9123827	-0.4000000	2.719492
3	4-1	2.4700590	0.4000000	3.900000
4	3-2	0.3824933	-0.2497459	1.049746
5	4-2	2.2078922	0.6000000	2.719492
6	4-3	1.8369234	0.4000000	2.449746

Tibia magnesium

	Comparison	Mean difference	95% CI_lower bound	95% CI_upper bound
1	2-1	0.02980183	-0.010000000	0.0850000
2	3-1	0.04536266	-0.015000000	0.1199831
3	4-1	0.10173158	0.009350270	0.1700000
4	3-2	0.01170335	-0.025000000	0.0500000
5	4-2	0.07930803	0.026713243	0.1200000
6	4-3	0.06807882	0.003333333	0.1250000

SUPPLEMENTARY FIGURES

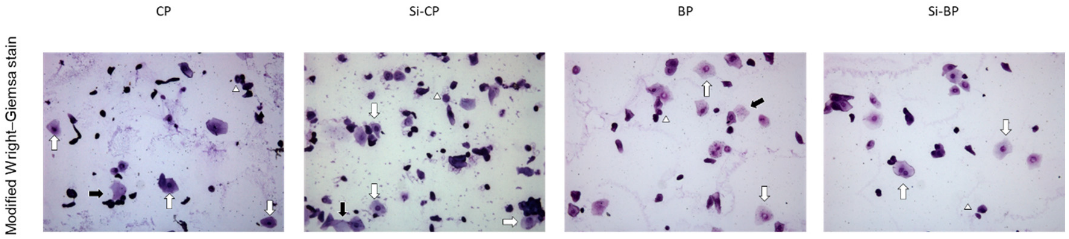

Figure S1. Representative images of vaginal smears. ⇨ Nucleated epithelial cells. ➡ Cornified epithelial cells. △ Lymphocytes.

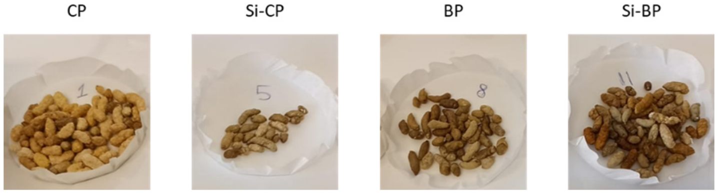

Figure S2. Representative images of daily fecal excretion.
